# The flavonoid rutin protects against imidacloprid-induced osmotic and electric disruptions in Africanized honey bees

**DOI:** 10.1371/journal.pone.0331855

**Published:** 2025-09-09

**Authors:** Juan P. Hernández, Fredy Mesa, Andre J. Riveros

**Affiliations:** 1 Departamento de Biología, Escuela de Ciencias e Ingeniería, Universidad del Rosario, Bogotá, Colombia; 2 Facultad de Ingeniería y Ciencias Básicas, Fundación Universitaria Los Libertadores, Bogotá, Colombia; 3 Department of Neuroscience, College of Science, University of Arizona, Tucson, Arizona, United States of America; Tanta University Faculty of Science, EGYPT

## Abstract

Honey bees (*Apis mellifera*) are essential pollinators threatened by sublethal effects of pesticides such as imidacloprid, a widely used neonicotinoid that disrupts the central nervous system. However, many of the systemic effects are poorly understood, especially on the physiological homeostasis of the honey bee. We evaluated the effects of oral administration of imidacloprid and the flavonol rutin on the properties of extracellular fluid (ECF) in *Apis mellifera*. We measured water content, evaporation rate, electrical impedance, and ion mobility of the ECF. Our results show impacts of imidacloprid consumption, such as water content decrease, slowed evaporation, and altered electrical characteristics of the thorax segment. All these events suggest disruption of osmotic and electrochemical balance. Particularly, the rutin consumption partially mitigated the imidacloprid effects in a dose-dependent manner, enhancing detoxification. Our results point out that imidacloprid alters ionic and osmotic homeostasis beyond neural targets; and on the other hand, rutin may protect against these disruptions through physiological mechanisms beyond neuroprotection. These findings highlight new alternatives and evaluations for protecting pollinators via dietary strategies.

## Introduction

Wild and managed bees play a fundamental role in ecosystems, not just as pollinators of wild flora but also as silent workers behind much of the food humans and livestock depend on [1–6]. Yet, in recent decades, their numbers have dropped at an alarming rate [7,8]. This decline does more than reduce pollination, it chips away at biodiversity [9,10] and, in a broader sense, shakes the foundation of global food security [11–13]. Among the many factors behind this crisis, pesticide exposure stands out, particularly in intense monocultures where these chemicals are applied almost routinely [6,14–16]. Neonicotinoids, a class of insecticides praised for their broad-spectrum effectiveness [17,18], also cast a long shadow. Imidacloprid, a first-generation neonicotinoid patented 35 years ago [19], remains widely used. While its toxicity is well documented, many of its sublethal effects on bees continue to be a growing concern [20–22].

The sublethal effects of imidacloprid can interfere with a range of biological functions. As a partial agonist of cholinergic nicotinic receptors [23–25], the compound primarily targets neurons in the insect brain [26,27]. This interaction alters sodium channel conductance, which in turn disrupts the delicate balance of the membrane potential [28,29]. It is hardly surprising that bees exposed to the insecticide often show impaired brain functions, where learning and memory seem to suffer [30–32], and so do motor control and navigation [33–36]. For example, studies have reported that even a dose as low as 20ng per bee may trigger tremors and loss of motor coordination, signs that neural signaling is taking a serious hit [33,35,37].

An idea that has been gaining traction to counter the impact of imidacloprid, and similar insecticides, is something researchers call the precision nutrition approach [38–42]. The concept is straightforward: supply bees with specific molecules that may offer prophylactic protection and boost their resilience [43–45]. It turns out that nutrition plays a bigger role than many expect. Diets rich in antioxidants and anti-inflammatory agents appear to help bees keep their physiological systems running under stress [46,47]. Plant-derived secondary metabolites have drawn attention here, as they seem to improve tolerance against toxic exposures [48–50]. Previous studies suggest that rutin can protect cognitive functions, not only in the bumble bee *Bombus impatiens* but also in the Africanized honey bee (*Apis mellifera*) exposed to imidacloprid and even fipronil [51].

The reported protection offered by rutin and similar plant metabolites raises more questions than it answers. What are these compounds really doing at the physiological level? Their action may not be limited to neurons alone. When insecticides disrupt membrane conductance [52,53], the distribution of ions can become skewed, and this imbalance might ripple out beyond local sites to affect systemic electrical conditions. How far this disturbance may extend remains unclear. Ions like Na+, K+ and Cl^-^ are more than just players in neural signaling; they also act as osmolytes, meaning any shift in their conductance can easily spill over into osmotic balance. This raises a pressing question: could these secondary metabolites help restore not only electrical properties but also the osmotic stability of bee tissues?

Thus, we explore these questions by examining how the extracellular fluid (ECF) of the Africanized honey bee (*Apis mellifera*) responds to imidacloprid, rutin, or both. The ECF is an ideal target because it surrounds tissues and internal organs, acting as a dynamic environment where nutrients, gases, and waste are constantly exchanged [54–58]. The composition and volume of this fluid contribute to internal balance by regulating water and solute levels [59–62]. Moreover, specialized organs such as Malpighian tubules and rectal glands regulate extracellular composition by adjusting water and ion excretion or reabsorption [63–67], allowing insects to maintain optimal homeostasis in response to environmental changes [68–72]. Thus, similar to a vertebrate blood test, analyzing the extracellular fluid may provide insights into changes in the overall physiological state. We deliberately focused on the ECF of the thorax. The abdomen was excluded to avoid the variability introduced by crop contents. The thorax, with its greater rigidity, was better suited for our standardized setup. This configuration allowed us to reliably measure electrical properties while remaining minimally invasive compared to approaches involving the head.

Our goal was fourfold. First, we investigated whether *ad libitum* administration of low and high doses of imidacloprid and rutin would differentially influence osmotic balance, as reflected by changes in water content. Second, we analyzed possible alterations in the colligative properties of the extracellular fluid, focusing on evaporation dynamics. Third, we assessed modifications in resistance and reactance under different treatments with sucrose, rutin, and imidacloprid. Finally, we examined solute electrical mobility to gain insight into the electrical behavior of dissolved ions.

Based on the known toxicodynamics of imidacloprid as a partial agonist of Na+/Ca^2^+ channels and its chemical properties, we expected that the enhanced inward cation movement could lower resistance and reactance in the ECF. However, previous studies suggest that pesticide exposure may also alter ion distribution through mechanisms unrelated to receptor activation, including indirect effects on membrane integrity. While our data do not directly address such processes, they prompted us to consider that imidacloprid could modify the extracellular environment in ways that produce an opposite electrical pattern. Regarding rutin, we anticipated that its presence would mitigate these alterations, yielding values closer to those observed in control bees.

Our findings point to clear dose-dependent effects of both rutin and imidacloprid. What stood out is how their influences seemed to pull in opposite directions across nearly every measured parameter. Bees that consumed imidacloprid tended to show signs of dehydration, a faster evaporation rate, and an increase in electrical resistance. In contrast, those receiving rutin retained more water, evaporated fluid more slowly, and displayed lower resistance values. These patterns appear to support the idea that rutin’s protective role may, at least partly, involve mechanisms that help maintain osmotic balance, although other factors could also be at play.

## Results

### Estimation of dosages

We found that the volume ingested by bees varied depending on the administered treatment (ANOVA: F_6;77_ = 103.374; p < 0.0001; Table 1). We found differences in the volume of solutions ingested, with higher values in groups exposed to rutin (0.6Ru, 0.3Ru) compared to those treated with imidacloprid (0.6Im, 0.3Im) or combinations of both compounds (0.6Im + 0.6Ru, 0.3Im + 0.3Ru). In particular, bees in the 0.6Ru treatment consumed the highest volume (mean±s.e.m. = 55.2 ± 6.2 µL/bee; Table 1), while those in the 0.6Im group exhibited the lowest consumption (mean±s.e.m. = 24.2 ± 1.9 µL/bee; Table 1). The acceptance of the solutions varied depending on the treatment composition, which influenced the effective exposure to each compound. The administered dose, calculated in ng/bee, reflected consumption differences, with the highest concentrations found in groups treated with rutin alone (0.6Ru: 33.1 ± 3.7ng/bee; Table 1), whereas the lowest values were observed in the high concentration imidacloprid treatment (0.3Im: 8.3 ± 0.8ng/bee; Table 1).

**Table 1:** Estimated consumption and doses across all experimental groups.

Treatment	N	Estimated volume (µL) consumed per bee (mean±s.e.m.)	Estimated dose (ng) per bee (mean±s.e.m.)
0.6Im	415	24.2 ± 1.9	14.5 ± 1.1
0.3Im	415	27.7 ± 2.6	8.3 ± 0.8
0.6Ru	424	55.2 ± 6.2	33.1 ± 3.7
0.3Ru	404	44.6 ± 3.6	13.4 ± 1.1
0.6Im + 0.6Ru	401	31 ± 2.1	18.6 ± 1.3
0.3Im + 0.3Ru	423	38 ± 4	11.4 ± 1.2
Control	418	41.3 ± 3.4	–

Data is presented as mean±s.e.m.

### Evaluation 1: wet and dry mass of thorax

**Fig 1:**
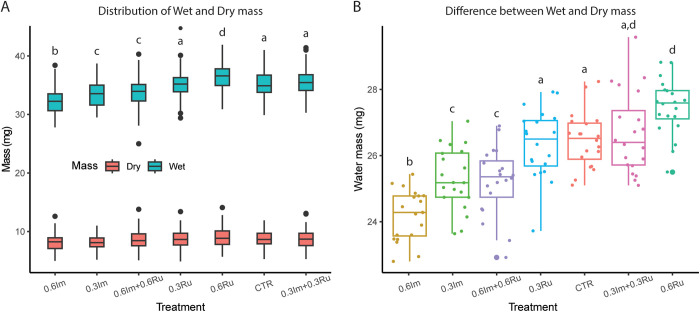
Effect of imidacloprid and rutin administration on wet mass, dry mass, and water content of the thorax in Africanized honey bee foragers. (A) Wet mass (blue bars) and dry mass (red bars) of thoraxes dissected after 24 h of ad libitum feeding on the assigned treatments. (B) Estimated water content calculated as the difference between wet and dry mass. Treatments include control (Ctr), imidacloprid (Im) at two concentrations (0.3 and 0.6 ppm), rutin (Ru) at two concentrations (0.3 and 0.6 ppm), and their combinations. Bars represent mean values ± SEM. Different letters above bars indicate statistically significant differences among treatments (ANOVA followed by Tukey’s HSD, p < 0.05).

We weighed 100 bees per treatment. We found that the administration of imidacloprid or rutin to bees led to statistically significant dose-dependent and contrasting effects relative to the administration of sucrose in the Control group (ANOVA: F_6,693_ = 41.10, p < 0.0001; Fig 1A). Bees receiving imidacloprid exhibited significantly lower wet masses (mean±s.e.m: m_w0.3Im_=33.4 ± 2.2 mg; m_w0.6Im_=32.3 ± 2.1 mg) than bees in the Control group (mean±s.e.m: m_wControl_ = 35.16 ± 2.23 mg; Control vs. 0.3Im *post hoc* Tukey test: 95%CI: −2.6, −0.8 mg, p < 0.0001; Control vs. 0.6Im: *post hoc* Tukey test: 95%CI: −3.8, −1.95 mg, p < 0.0001).

Conversely, bees receiving rutin exhibited wet masses that did not significantly differ from bees in the Control group (mean±s.e.m: m_w0.3Ru_=35.1 ± 2.2; Control vs. 0.3Ru: *post hoc* Tukey test: 95%CI = −1.0, 0.9 mg, p = 0.98; Fig 1A) or exhibited significantly higher mass when the rutin concentration was increased (mean±s.e.m: m_w0.6Ru_=36.5 ± 2.3 mg; Control vs. 0.6Ru: *post hoc* Tukey test: 95%CI = 0.4, 2.3 mg, p < 0.0001; Fig 1A). Interestingly, bees receiving the low dose mixture (0.3Ru + 0.3Im) did not exhibit significant differences in wet mass relative to bees in the Control group (mean±s.e.m: m_w0.3Ru+0.3Im_ = 35.4 ± 2.3 mg; Control vs. 0.3Ru + 0.3Im: *post hoc* Tukey test: 95%CI = −0.7, 1.2 mg, p = 0.98; Fig 1A). However, bees receiving the high dose mixture (0.6Ru + 0.6Im) exhibited significant differences in wet mass relative to bees in the Control group (mean±s.e.m: m_w0.6Ru+0.6Im_ = 33.8 ± 2.3 mg; Control vs. 0.6Ru + 0.6Im: *post hoc* Tukey test: 95% CI = −2.3, −0.44 mg, p < 0.0001; Fig 1A).

When assessing water balances (m_w_-m_D_), we also found dose and molecule dependence in the significant differences between the bees exposed to different treatments (F_6,133_ = 26, p < 0.0001). Bees receiving imidacloprid exhibited decreases at low (Control vs. 0.3Im: *post hoc* Tukey test: 95%CI = −2.1, −0.3 mg, p < 0.003; Fig 1B) and high concentrations relative to bees in the Control group (Control vs. 0.6Im: *post hoc* Tukey test: 95%CI = −3.2, −1.4 mg, p < 0.0001; Fig 1B). In contrast, bees receiving rutin either maintained (Control vs. 0.3Ru: *post hoc* Tukey test = 95%CI: −1.0, 0.8 mg, p = 0.98; Fig 1B) or increased the water content relative to bees in the Control group (Control vs. 0.6Ru: *post hoc* Tukey test = 95%CI: 0.08, 1.9 mg, p < 0.02; Fig 1B). Interestingly, bees receiving the mixtures exhibited a dose-dependent response (Control vs. 0.3Ru + 0.3Im: *post hoc* Tukey test: 95%CI = −0.7, 1.1 mg, p = 0.98; Control vs. 0.6Ru + 0.6Im: *post hoc* Tukey test: 95%CI = −2.2, −0.3 mg, p < 0.0001; Fig 1B).

### Evaluation 2: extracellular fluid evaporation

**Fig 2:**
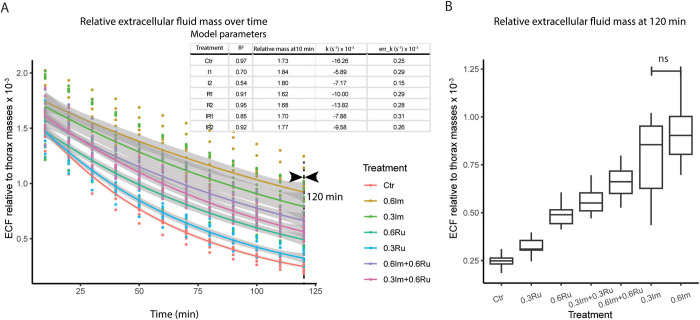
Effect of imidacloprid (Im) and rutin (Ru) administration on evaporation dynamics of extracellular fluid (ECF) extracted from thoraxes of Africanized honey bee foragers. (A) Evaporation curves of ECF showing mass loss (relative mass, ECF mass/ pooled thorax mass) over time (minutes) at 35°C for each treatment. The upper panel summarizes the fitted model parameters, including correlation coefficients (R²), relative mass, and evaporation constant (k). (B) Final ECF evaporation measured at 120 minutes. Treatments include control (Ctr), imidacloprid (Im) at 0.3 and 0.6 ppm, rutin (Ru) at 0.3 and 0.6 ppm, and their combinations. Lines represent mean values, and shaded areas denote SEM. Different letters above bars in panel B indicate statistically significant differences among treatments (Kruskal–Wallis test, p < 0.05).

We collected and processed 100 bees per treatment. We found that the evaporation curves followed an exponential trend that varied across treatments. Bees receiving only sucrose (Control) had an ECF with an evaporation trend that closely fit an exponential model with a low evaporation constant [*k*=(−16.26 ± 0.25)x10^-3^; R²=10.97]. Importantly, the administration of imidacloprid led to a dose-dependent decrease in the evaporation rate (0.3Im: *k*=(−5.73 ± 0.29)x10^-3^; 0.6Im: k=(−7.03 ± 0.15)x10^-3^). In contrast, when administered with rutin, ECF of bees exhibited evaporation rates that more closely resembled those of bees in the Control group (0.3Ru: *k*=(−10.00 ± 0.29)x 10^-3^; 0.6Ru: *k*=(−13.82 ± 0.28)x10^-3^). Finally, when bees received the combination, their ECF exhibited intermediate values (0.3Ru+0.3Im: *k*=(−7.88 ± 0.31)x10^-3^; 0.6Ru+0.6Im: *k*=(−9.58 ± 0.26)x10^-3^; Fig 2A).

Statistical comparisons between treatments at minute 120 further supported the pattern observed in the mass of ECF samples relative to the thorax mass from which they were extracted (Kruskal-Wallis: 120 min: χ²₆ = 58.05, p < 0.0001; Fig 2B). In all cases, the administration of imidacloprid, rutin, or their combinations resulted in a lower evaporation rate. The ECF of bees exposed to imidacloprid remained in the highest quantity at the end of the 120-minute period (mean±SD: 0.6Im = 0.92 ± 0.18; 0.3Im = 0.79 ± 0.22). Similarly, rutin consumption led to fluid retention levels higher than those observed in the Control group (mean±SD: Control = 0.24 ± 0.04; 0.6Ru = 0.49 ± 0.06; 0.3Ru = 0.32 ± 0.04).

### Evaluation 3: electrical impedance analysis

**Fig 3:**
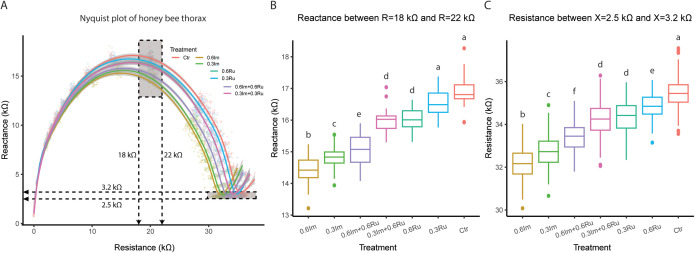
Effect of imidacloprid (Im) and rutin (Ru) administration on thoracic electrical impedance in Africanized honey bee foragers. (A) Nyquist plots showing reactance (kΩ, imaginary component) as a function of resistance (kΩ, real component) for the thorax under different treatments. Curves illustrate treatment-dependent variations in electrical properties, where a rightward shift reflects increased extracellular resistance and larger semicircle radii indicate higher tissue capacitance. (B) Comparison of resistance values within the 18–22 kΩ range, highlighting differences in reactance among treatments. (C) Comparison of resistance in the 2.5–3 kΩ range, showing variations in electrical resistance. Bars represent mean ± SEM, and different letters above treatments indicate statistically significant differences (ANOVA followed by Tukey’s HSD, p < 0.05).

We collected, prepared, and processed 50 bees per treatment. The impedance exhibited by the bees showed both capacitive and resistive behavior, as evidenced in the Nyquist plots. The electrical properties of the thorax ECF were influenced by the presence of both charged and uncharged molecules; charged species contributed to ionic conduction and uncharged species affected polarization effects. Charged molecules exhibited mobility due to the applied potential difference, contributing to ionic conduction, whereas uncharged molecules underwent polarization due to the same potential, affecting capacitive behavior. The Nyquist plots revealed semicircular impedance behavior, where the resistive component reflects ion mobility in the extracellular fluid, and the capacitive component represents charge storage at membrane interfaces (Fig 3A).

Thorax reactance, measured at resistance values between 18kΩ and 32kΩ, exhibited significant differences across treatments (F_6,168_ = 107.2, p < 0.0001; Fig 3B). In the bees within the Control group, the mean reactance in this resistance range was 16.9 ± 0.5kΩ, whereas lower values were observed in all treated groups. Bees exposed to high-concentration of imidacloprid (0.6Im) exhibited a 2.4kΩ reduction in reactance relative to bees in the Control group (*post hoc* Tukey test = 95%CI: −2.8, −2.1kΩ, p < 0.0001; Fig 3B), while the exposure to low-concentration (0.3Im) reduced reactance by 2.0kΩ (*post hoc* Tukey test = 95%CI: −2.4, −1.7kΩ, p < 0.0001; Fig 3B). In contrast, bees administered with the low concentration of rutin (0.3Ru) did not exhibit significant changes in reactance relative to bees in the Control group (*post hoc* Tukey test = 95%CI: −0.3, 0.7kΩ, p = 0.06; Fig 3B). Nevertheless, bees receiving high concentration rutin (0.6Ru) decreased their reactance by 0.8kΩ (*post hoc* Tukey test = 95%CI: −1.1, −0.8kΩ, p < 0.0001; Fig 3B). The administration of the mixture of imidacloprid and rutin resulted in intermediate effects: the high-dose composition (0.6Ru + 0.6Im) reduced reactance by 1.8kΩ (95%CI: −2.1, −1.4kΩ, p < 0.0001; Fig 3B), while the low-dose composition decreased by only 0.8kΩ (95%CI: −1.2, −0.7kΩ, p < 0.0001; Fig 3B).

Thorax resistance, measured at reactance values between 2.5kΩ and 3.2kΩ, also exhibited significant treatment-dependent differences (ANOVA: F_6,1510 _= 627.7, p < 0.0001; Fig 3C). Bees in the Control group had a mean resistance of 35.5 ± 0.7kΩ (mean±s.e.m), while lower values were observed in all treated groups. Bees exposed to high-concentration imidacloprid (0.6Im) exhibited a 3.3kΩ decrease in resistance (*post hoc* Tukey test = 95%CI: −3.5, −3.1kΩ, p < 0.0001; Fig 3C), whereas the exposure to low-concentration (0.3Im) reduced resistance by 2.7kΩ (*post hoc* Tukey test = 95%CI: −3.0, −2.6kΩ, p < 0.0001; Fig 3C). The administration of a low-concentration of rutin (0.3Ru) led to a decreased resistance by 1.1kΩ compared to the bees in the Control group (*post hoc* Tukey test = 95%CI: −1.3, −0.9 kΩ, p < 0.0001; Fig 3C), while the administration of high-concentration rutin (0.6Ru) reduced resistance by 0.6kΩ (*post hoc* Tukey test = 95%CI: −0.8, −0.4 kΩ, p < 0.0001; Fig 3C). Similar to the effects on reactance, the administration of the compositions of imidacloprid and rutin produced intermediate effects: the high-dose combination (0.6Ru + 0.6Im) reduced the resistance by 2.0kΩ (*post hoc* Tukey test = 95%CI: −2.2, −1.8kΩ, p < 0.0001; Fig 3C), while the administration of the low-dose composition (0.3Ru + 0.3Im) resulted in a 1.2kΩ reduction (*post hoc* Tukey test = 95%CI: −1.3, −1.1 kΩ, p < 0.0001; Fig 3C).

### Evaluation 4: current-voltage (I-V) curves

**Fig 4:**
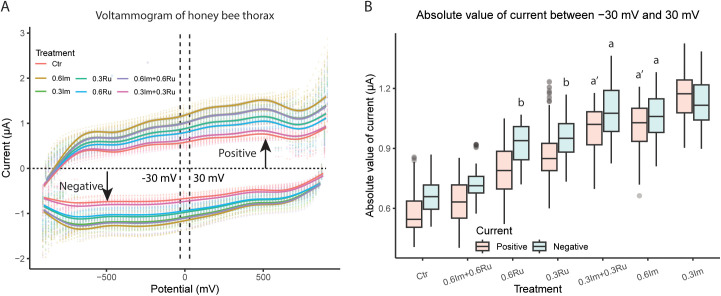
Effect of imidacloprid (Im) and rutin (Ru) administration on thoracic electric currents in Africanized honey bee foragers. (A) Voltammograms showing the current response as a function of applied potential (−800 to 800 mV) for each treatment. The slope of the I–V curve reflects ionic conductivity, while deviations from linearity indicate capacitive contributions of thoracic tissue. (B) Absolute values of current recorded within the −30 to 30 mV range, where both positive and negative components of current were quantified separately to compare treatments. Treatments include control (Ctr), imidacloprid (Im) at 0.3 and 0.6 ppm, rutin (Ru) at 0.3 and 0.6 ppm, and their combinations. Bars represent mean ± SEM, and different letters above treatments indicate statistically significant differences (ANOVA followed by Tukey’s HSD, p < 0.05). Only significant comparisons are displayed to improve visualization.

We collected, prepared, and processed 50 bees per treatment to evaluate the I-V characteristics of the thorax extracellular fluid. The electrical response exhibited both resistive and non-ohmic behavior, confirming the presence of charge transporting mechanisms influenced by ionic mobility and interface polarization. The applied potential generated a nonlinear I-V relationship, where charged species in the extracellular fluid contributed to current flow due to electrophoretic forces, while uncharged molecules exhibited capacitive effects due to dielectric polarization. The asymmetric shape of the I-V curves confirmed that charge redistribution and membrane-associated electrochemical interactions played a role in the electrical response (Fig 4A).

The electrical current exhibited significant differences among treatments in the thorax’s electrical response under ascending and descending voltages. The recorded currents increased or decreased according to the applied potential, displaying non-ohmic behavior. The current across all treatments was different from zero at zero applied voltage, indicating additional electrochemical components in the system, such as polarization effects or capacitive contributions from the extracellular fluid. This deviation from pure ohmic behavior suggests that electrical conduction is governed both by the fluid’s resistive properties and charge accumulation at electrode interfaces (Fig 4A). Within the voltage range of -30mV to 30mV, absolute current values exhibited similar trends for both ascending and descending voltage conditions, indicating that charge mobility was influenced by the applied electric field orientation. The current measured through the thorax in this voltage range showed significant treatment-dependent differences for both increasing (ANOVA: F₆,₁₀₀₁ = 471.16, p < 0.0001) and decreasing (ANOVA: F₆,₁₀₀₁ = 412.03, p < 0.0001) voltages.

Bees that consumed imidacloprid exhibited the highest currents, indicating a greater presence of mobile ions in the extracellular fluid (0.6Im vs. Control: *post hoc* Tukey test, 95%CI: 0.55, 0.63µA, p < 0.0001; 0.3Im vs. Control: *post hoc* Tukey test, 95%CI: 0.43, 0.39µA, p < 0.0001). The consumption of rutin also increased electrical currents in the bees compared to the bees in the Control group, but to a lesser extent than imidacloprid (0.6Ru vs. Control: *post hoc* Tukey test, 95%CI: 0.30, 0.26µA, p < 0.0001; 0.3Ru vs. Control: *post hoc* Tukey test, 95%CI: 0.21, 0.17µA, p < 0.0001). A remarkable observation was that when bees consumed 0.3Im + 0.3Ru, the values of currents were closer to those exhibited by the bees within the Control group (0.3Im + 0.3Ru vs. Control: *post hoc* Tukey test, 95%CI: 0.06, 0.02µA, p < 0.0001; Fig 4B).

## Discussion

The impairments caused by pesticide and pollutant exposure remain a major concern for the pollination services provided by bees. Insecticides such as neonicotinoids target neural receptors and have been associated with sublethal impairments in cognitive functions, including learning, memory, navigation, and motor control. However, other systemic effects are still not fully understood. Here, we aimed to determine whether the oral administration of imidacloprid could alter the electrochemical properties of the extracellular fluid (ECF). Additionally, we tested whether rutin, a flavonol known for its potential protective effects against insecticides, might mitigate the impact of imidacloprid. Our results indicate that, relative to control conditions, imidacloprid exposure generally caused dose-dependent decreases in water content, ECF evaporation rate, electrical reactance, and resistance, while increasing electrical currents. In contrast, rutin administration, either alone or in combination with imidacloprid, resulted in values closer to those observed in control bees. These findings suggest three main aspects: (i) a substantial alteration in the extracellular electrochemical environment caused by imidacloprid, (ii) dose-dependent changes in response to imidacloprid and/or rutin likely due to their chemical properties and associated physiological actions, and (iii) partial amelioration of imidacloprid’s impact when co-administered with rutin.

First, our results indicate that imidacloprid-induced ECF alterations may extend beyond receptor activation. Like other neonicotinoids, imidacloprid is known to affect membrane ion conductance. As a partial agonist of nicotinic acetylcholine receptors (nAChR), it is expected to enhance inward cationic currents (Na ⁺ , Ca²⁺) [73,74], which theoretically could increase ECF resistance. However, we observed a decrease in both resistance and reactance, along with higher electrical currents, which may suggest an increase in free charged particles within the ECF. This pattern is consistent with the possibility of membrane leakage or damage caused by the 24 h ad libitum exposure to the insecticide. Imidacloprid is known to induce oxidative stress, leading to the production of reactive oxygen and nitrogen species (ROS and RNS, respectively) [75–77]. Evidence from zebrafish [78], earthworms [79], mice [80] and rats [81,82] indicates that physiological responses to ROS and RNS can enhance lipid peroxidation, as reflected by elevated levels of malondialdehyde and thiobarbituric acid-reactive substances, both major byproducts of lipid oxidation. It is plausible that oxidative damage destabilizes membrane lipids, increasing ion leakage and thereby contributing to the reduction in resistance and reactance, as well as to the observed increase in current. Such disruption could impair muscle and nerve function, potentially affecting motor coordination and responsiveness.

Second, we observed a non-linear dose-dependent effect caused by the administration of imidacloprid (0.3Im vs. 0.6Im), rutin (0.3Ru vs. 0.6Ru), and their combination (0.3Im + 0.3Ru vs. 0.6Im + 0.6Ru). This non-linearity could be partly attributed to variations in self-administered doses. Doubling the concentration of imidacloprid led to an approximate 9% decrease in liquid consumption, resulting in only a 0.75-fold increase in the administered dose (Table 1). In contrast, doubling the rutin concentration increased ingestion by around 21%, leading to a 2.5-fold increase in dose. Interestingly, the combined administration of imidacloprid and rutin resulted in intermediate consumption behavior, with a 1.6-fold increase in dose. These patterns may arise from non-mutually exclusive mechanisms. Imidacloprid might induce a satiation-like effect, explaining the ~ 42% reduction in consumption relative to control bees (Table 1), whereas rutin could have the opposite influence, consistent with the ~ 34% increase in ingestion observed. Alternatively, imidacloprid may impair crop emptying, mechanically restricting further intake, while rutin could enhance thirst or reduce satiety signals.

Despite these differences in consumption, the physiological effects observed were consistent with dose-dependent responses. The reduction in water content caused by imidacloprid increased from ~5% (0.3Im) to ~8% (0.6Im) relative to control bees. Conversely, rutin administration led to greater water retention, increasing from ~0% (0.3Ru) to 4% (0.6Ru) above control levels. These differences are unlikely to be solely explained by ingestion variations, as the changes in water content did not scale directly with consumption. Instead, they may reflect intrinsic properties of rutin as an osmolyte, its potential to stabilize membranes [82,83], or the upregulation of aquaporins, a mechanism previously demonstrated in rats [84]. Dose-dependent effects were also evident in ECF evaporation rates (Fig 2). Imidacloprid significantly reduced extracellular fluid evaporation compared to the control group, which may indicate compositional changes that increase viscosity or solute concentration. From an ecological perspective, disturbances in water balance could impair thermoregulation, potentially making bees more vulnerable to dehydration and adverse climatic conditions [85–87].

Third, we observed that the co-administration of imidacloprid and rutin generally mitigated several of the negative effects associated with imidacloprid. For example, bees receiving 0.6Im showed an approximate 8% reduction in water content relative to controls, while the addition of 0.6Ru (0.6Im + 0.6Ru) appeared to reduce this loss by about 50% (Table 1). Likewise, administration of 0.3Im alone resulted in a ~ 5% reduction in water content, whereas the combination with 0.3Ru (0.3Im + 0.3Ru) prevented this decrease almost entirely. Similar patterns of amelioration were detected in ECF evaporation rates (Fig 2). These protective effects may be linked to rutin’s influence on osmoregulatory processes, possibly through modulation of Malpighian tubule activity [67,70], which is known to play a central role in insect excretion and water balance [68]. The enhanced ECF evaporation rate observed in rutin-treated groups could indicate that this flavonoid helps stabilize hemolymph composition while supporting water retention.

**Fig 5:**
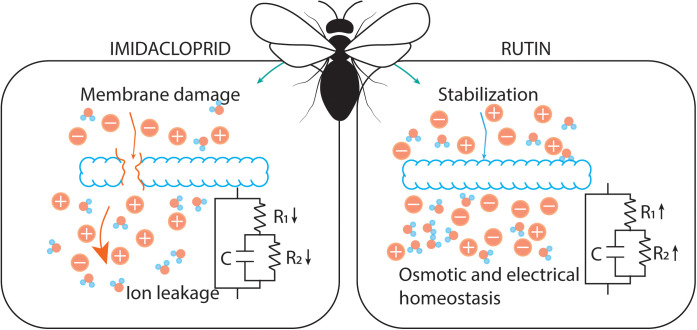
Diagram summarizing the physiological effects of imidacloprid and rutin on extracellular ionic and osmotic balance in Africanized honey bees (*Apis mellifera*). The diagram illustrates how imidacloprid exposure may destabilize membrane integrity, leading to ion leakage, reduced electrical resistance and reactance, and decreased water content in thoracic tissues. These alterations potentially impair motor and neural functions by disrupting osmotic and electrical homeostasis. In contrast, rutin administration is depicted as contributing to membrane stabilization, improved ion regulation, and enhanced water retention, which may counteract the negative effects of imidacloprid. Together, these pathways highlight the opposing physiological actions of the two compounds on bee extracellular fluid properties.

Additionally, the concurrent administration of rutin attenuated the imidacloprid-induced changes in resistance, reactance, and electrical current. These observations are consistent with the hypothesis that rutin contributes to membrane stabilization and improved ion regulation [87–90]. Competitive or allosteric interactions between rutin and imidacloprid at receptor level may also play a role, although further studies are needed to confirm this mechanism. Moreover, rutin, similar to other flavonoids, may influence detoxification pathways, such as the cytochrome P450 enzyme family [44,90], potentially enhancing insecticide metabolism. While these possibilities remain speculative, they provide plausible explanations for the observed protective effects. Importantly, previous studies have primarily evaluated prophylactic administration of rutin [51,91], whereas our results suggest that protection may also occur during concurrent exposure, highlighting rutin’s potential as a mitigating agent under realistic exposure scenarios (Fig 5).

Despite its protective effects, rutin alone also induced some dose-dependent physiological alterations relative to control bees. Higher rutin dosage (0.6Ru) was associated with increased water retention, reduced ECF evaporation rates (0.3Ru, 0.6Ru), decreased resistance (0.3Ru, 0.6Ru), decreased reactance (0.6Ru), and increased electrical currents (0.3Ru, 0.6Ru). Although no behavioral or physiological impairments were detected in bees receiving rutin in our previous or current experiments, it is important to consider that rutin is a plant-derived secondary metabolite originally evolved to deter herbivores and insects [92–94]. Therefore, some degree of physiological stress may be expected as part of its action.

We have previously proposed that rutin’s protective effects could involve hormetic responses [51,91], where low levels of stress trigger adaptive mechanisms that enhance resistance to insecticides. The present results are consistent with this idea, as the physiological changes induced by rutin alone did not appear detrimental and may instead prime bees to better cope with imidacloprid exposure. However, confirming this mechanism will require future experiments explicitly designed to test hormesis and its relationship to ion regulation and membrane stability.

Even though our experiments point to clear patterns in how imidacloprid disrupts bee physiology and how rutin might mitigate those effects, there are things we need to keep in mind. For one, the work was done in a lab with bees from just a few colonies, so it probably doesn’t capture the messy reality of field conditions where many stressors act at once. Also, we only looked at the thorax and its extracellular fluid, meaning other tissues and systemic effects remain a question mark. When it comes to the electrical measurements, they gave us a new perspective, but part of what we infer still needs biochemical or molecular backing to be sure. And lastly, our tests with rutin were under controlled co-exposure; how this plays out when bees forage freely in the wild is still an open question. These gaps are not deal breakers; instead, they set the stage for the next steps, where field work and deeper analyses could reveal how far these findings truly extend.

Our findings suggest that imidacloprid disrupts water homeostasis and the electrochemical balance in Africanized honey bees, likely compromising tissue function and extracellular fluid stability. Such alterations may reduce resilience to environmental stressors, impair foraging efficiency, and interfere with navigation. Co-administration of rutin appeared to mitigate several of these negative effects, pointing to its potential as a protective compound under pesticide exposure. From an ecological perspective, these results open the possibility that dietary flavonoids could enhance pollinator resilience, although field validations are still needed to confirm this protective role under natural conditions.

These observations also emphasize the importance of developing mitigation strategies to safeguard pollinators from pesticide-induced harm, while reinforcing the need for tighter regulations to minimize sublethal stress in bees. Beyond their applied implications, the physiological principles explored here, including ion regulation, osmotic balance, and electrical conductance, are broadly conserved across insect taxa. Therefore, the experimental framework presented in this study may be adapted to other species, such as Drosophila melanogaster or Bombus terrestris, enabling comparative analyses of systemic responses to environmental toxins. Ultimately, this approach offers a promising avenue for advancing insect physiology and ecotoxicology research while highlighting the value of electrical and osmotic markers as early indicators of stress in pollinators.

## Materials and methods

### Experimental animals and conditions

We collected adult foragers of the Africanized honey bee *Apis mellifera* from hives located at the apiary of the Universidad del Rosario (Bogotá, Colombia). The bees were captured in disposable plastic cups (9 oz) equipped with feeders containing a 1M sucrose solution. Immediately after capture, the bees were transported to the laboratory and maintained for 24h in an incubator (T = 35°C; RH = 55%). At the end of this acclimation period, the bees were administered according to the assigned experiment with the insecticide imidacloprid, the flavonol rutin, or their combination (see below). In all cases, the compositions were dissolved in a 1M sucrose solution to encourage consumption.

This research did not require approval from an institutional animal care committee, as honey bees are invertebrates and are not subject to animal experimentation regulations in Colombia. Nevertheless, all handling and experimental procedures were conducted according to international guidelines to minimize stress and ensure the welfare of the bees.

### Imidacloprid and rutin dosage

We relied on a commercial form of imidacloprid (Confidor350, Bayer), aiming to test realistic conditions experienced by bees in crops. The imidacloprid concentrations were prepared to maintain sublethal concentrations based on reported LD_50_ data (the dose at which 50% of a test population is lethally affected). This concentration exceeded the reported in pollen (0.001 ppm; Jiang et al., 2018) or nectar (0.065 ppm; Jiang et al., 2018) in the field, but served as a critical challenge to test hypotheses of protection by the administration of rutin. Thus, we used two concentrations of the insecticide: 0.3 ppm (0.3Im), 0.6 ppm (0.6Im).

Rutin concentrations were prepared in the same magnitude order as imidacloprid, ensuring proportionality and functionality. Defining realistic concentrations is challenging because of the broad variation of rutin found in nectar and pollen (Guffa et al, 2017; Gullón et al. 2017). However, we have previously shown effectiveness of cognitive protection in bumble bees and honey bees using concentrations of 0.6 ppm [44,51]. Thus, we prepared two concentrations of rutin: 0.3 ppm (0.3Ru), 0.6 ppm (0.6Ru). Following these concentrations and the mixtures, groups of bees were randomly assigned to one of seven treatments: Control (1M sucrose water), 0.3Rut, 0.6Ru, 0.3Im, 0.6Im, 0.3Ru + 0.3Im, 0.6Ru + 0.6Im. The bees were maintained in the incubator (see conditions above) and allowed to drink the assigned composition *ad libitum* for 24h. Then, the bees were allocated to one of four evaluations.

Each treatment group consisted of 100 bees for wet and dry mass measurements and 50 bees for current-voltage (I–V) characterization. These sample sizes were determined based on the need to minimize variability while ensuring reliable measurements with our experimental setup. For the electrical evaluations, preliminary tests indicated that using at least three bees per measurement was necessary to obtain a detectable signal, and replicating measurements with multiple batches increased robustness. Although no previous studies have used this exact methodology, the chosen sample sizes provided sufficient data to identify differences between treatments.

All solutions were prepared fresh daily to prevent degradation. Rutin was dissolved directly in 1 M sucrose by gentle stirring until fully dissolved. Both rutin and imidacloprid solutions were stored at 4°C during short intervals of the experimental period and protected from light to minimize chemical breakdown.

### Evaluation 1: wet and dry mass of thorax

Following the exposure period, batches of 20 bees per treatment were sacrificed by freezing at −20°C for 24h. This extended freezing period minimized evaporation upon removal from freezer. Then, the bees were dissected to obtain the thorax. The wet mass of each thorax was measured using a precision scale (0.1 mg precision; Radwag AS 220.R2). The thoraxes were then placed in a drying oven at 70°C for 48h. After this period, the dry mass was recorded using the same scale. The difference between wet (m_W_) and dry mass (m_D_) was used to estimate body water content (water content = m_W_-m_D_).

### Evaluation 2: extracellular fluid evaporation

This evaluation aimed to identify changes in colligative properties of the extracellular fluid (ECF). After the exposure period, batches of 10 bees per treatment were sacrificed by freezing at −20°C for 24h. The bees were then removed from the freezer, and their thoraxes were dissected and weighted as pools according to treatment. The thoraxes were placed in pipette tips with filters to extract extracellular fluid by centrifugation (3000RCF, 5 min, 4°C). Samples of 0.5 mL of extracellular fluid were deposited into cell culture wells, and the initial mass of the wells was recorded (m_0_). The ECF samples were subjected to evaporation at 35°C for 2h, with mass (m) measurements recorded every 10 min. This process allowed characterization of evaporation properties for each treatment over time, with evaporation rates reflecting solute concentrations (mass = m-m_0_). The centrifugation procedure typically yielded between 0.4 and 0.6 mL of extracellular fluid per 10 thoraxes, consistent with our previously published extraction protocol [95].

### Evaluation 3: electrical impedance analysis

Electrical impedance (Z) describes the opposition of a biological sample to alternating current flow when a voltage difference is applied. Here, a parallel resistive-capacitive (RC) model was used, suitable for representing heterogeneous biological tissues such as the bee thorax, where extracellular fluid with high ion concentrations acts as a resistive conductor, while cell membranes function as capacitive elements due to their lipid bilayer structure. Mathematically, impedance is expressed as:


$$Z=\frac{R}{\text{1}+R^{\text{2}}\omega^{\text{2}}C^{\text{2}}}-\frac{R^{\text{2}}\omega C}{\text{1}+R^{\text{2}}\omega^{\text{2}}C^{\text{2}}}j$$

where R is the resistance of the extracellular fluid, and C is the capacitance from the cell membranes. Impedance (Z) consists of a real component (R), representing ionic conduction resistance in extracellular fluid, and an imaginary component (-X), representing capacitive reactance due to charge storage in cell membranes.

For our evaluation, we characterized the electrical properties of live bees connected to a capacitor and a resistor. Electrical measurements were performed using batches of three bees *per* treatment simultaneously to increase the electrical signal. Measurements were conducted using two stainless steel electrodes made of hypodermic needles (diameter = 0.18 mm) inserted into the thorax and arranged in parallel. This configuration was connected in series with a 224nF ceramic capacitor and a 2200 Ω resistor. Measurements were performed using an LCR meter (Keysight E4980AL), configured to measure resistance and reactance with an excitation potential of 300mV. The LCR meter was controlled using the Command Expert software from Excel to ensure measurement accuracy. The results were represented in Nyquist diagrams, plotting reactance versus resistance. A larger semicircle radius indicated higher tissue capacitance, while a rightward shift suggested increased extracellular fluid resistance, providing insights into changes in ionic composition and tissue structure under different treatments. The experiment was repeated five times. The LCR meter was calibrated using the manufacturer’s standard calibration procedure before each set of measurements to ensure accuracy and consistency.

### Evaluation 4: current-voltage (I-V) curves

Current (I)-voltage (V) curves (hereafter I-V curves) characterize biological samples by analyzing the current response to an applied voltage difference. In this study, an electrical model was applied to describe the bioelectrical behavior of the bee thorax, where the measured current is the sum of two main components:


$$I=C\frac{dV}{dt}+\frac{V}{R}$$

The first term, $C\frac{dV}{dt}$, represents capacitive current due to charge accumulation on cell membranes acting as natural capacitors. The second term, *V*/*R*, corresponds to resistive current from ionic conduction through extracellular fluid, particularly hemolymph rich in ionic solutes. Voltage was applied within a range of -800mV to 800mV at a 0.18mV/s rate, selected to evaluate physiological electrical behavior without inducing electroporation damage. The current-voltage relationship was represented in voltammograms, with voltage on the x-axis and current on the y-axis. Variations in ionic conductivity and tissue capacitance provided insights into solute composition and mobility in the thorax of bees exposed to different experimental treatments.

Electrical conductivity of the thorax was evaluated using I-V curves in live bees to assess the presence of electrically mobile solutes. Measurements were performed on three bees with two stainless steel electrodes inserted in the thorax (see Evaluation 3 for details). The bees were connected in series with a 2200 Ω resistor. Measurements were performed using a source measurement unit (Keithley 2400 Graphical Series SMU). The applied voltage ranged from -800mV to 800mV, with a change rate of 0.18mV/s. Data were filtered within the -30mV to 30mV range to compare absolute currents obtained across treatments. The full procedure for bee immobilization, electrode insertion, and electrical connection is shown in Supplementary Videos S1–S7 in S3 File, which detail each step of the preparation and measurement process. The source measurement unit (Keithley 2400) was calibrated according to the manufacturer’s specifications before each experimental session to minimize instrumental drift.

### Statistical analysis

The data obtained were analyzed using parametric and non-parametric tests depending on the distribution and sample size. For data that met the assumptions of normality and homogeneity of variances, an analysis of variance (ANOVA) was performed, followed by Tukey’s *post-hoc* tests to identify significant differences between experimental groups. When data did not meet these criteria or when sample sizes were small (n ≤ 35), non-parametric tests such as the Kruskal-Wallis test were applied, followed by pairwise Wilcoxon tests for *post-hoc* comparisons. A significance level of p < 0.05 was considered in all statistical analyses. Data processing, statistical analyses, and figure generation were performed using R statistical software (v.4.1.2). Data were imported and cleaned using the tidyverse package; statistical tests such as ANOVA, Tukey’s HSD, and Kruskal–Wallis were conducted using rstatix, and all plots were generated with ggplot2 and ggh4x. A detailed and reproducible R Markdown script, including all analysis steps and figure export commands, is provided as Supplementary S2 File.^‌‌^

## Supporting information

S1 File Supplementary File S1 contains the complete raw dataset, including individual measurements of thorax wet and dry mass, water loss, evaporation dynamics over time, impedance variables (resistance, reactance, impedance modulus), and current–voltage values for all experimental groups. (XLSX)

S2 File Supplementary File S2 provides the full R Markdown script used for data cleaning, statistical analyses, and figure generation related to mass, evaporation, impedance, and I–V curves, ensuring transparency and reproducibility of the analyses. (RMD)

S3 File Supplementary Videos (File S3) show a step-by-step protocol for preparing and connecting *Apis mellifera* workers to the electrical measurement system. The videos demonstrate bee stabilization using micropipette tips, thorax immobilization with parafilm, and the subsequent fixation, electrode insertion, and connection required to perform electrical measurements.
(M4V)
